# Supplementary material for: Total whole-arm chromosome losses predict malignancy in human cancer
Source: Proc Natl Acad Sci U S A. 2025 May 2;122(18):e2505385122. doi: 10.1073/pnas.2505385122 (PMC12067283; doi:10.1073/pnas.2505385122)
Supplement: Supplementary file 1 — Appendix 01 (PDF) [file pnas.2505385122.sapp.pdf]

PNAS

# Supporting Information for

Total whole-arm chromosome losses predict malignancy in human cancer

Ye Zheng[1,2], Kami Ahmad[1] and Steven Henikoff[1,3,*]

[1]Fred Hutchinson Cancer Center, Seattle, WA 98109
[2]Present address: University of Texas MD Anderson Cancer Center, Houston, TX
[3]Howard Hughes Medical Institute, Chevy Chase, MD 20815

*Steven Henikoff
**Email:** steveh@fredhutch.org

**This PDF file includes:**

Figures S1 to S4
Table S1

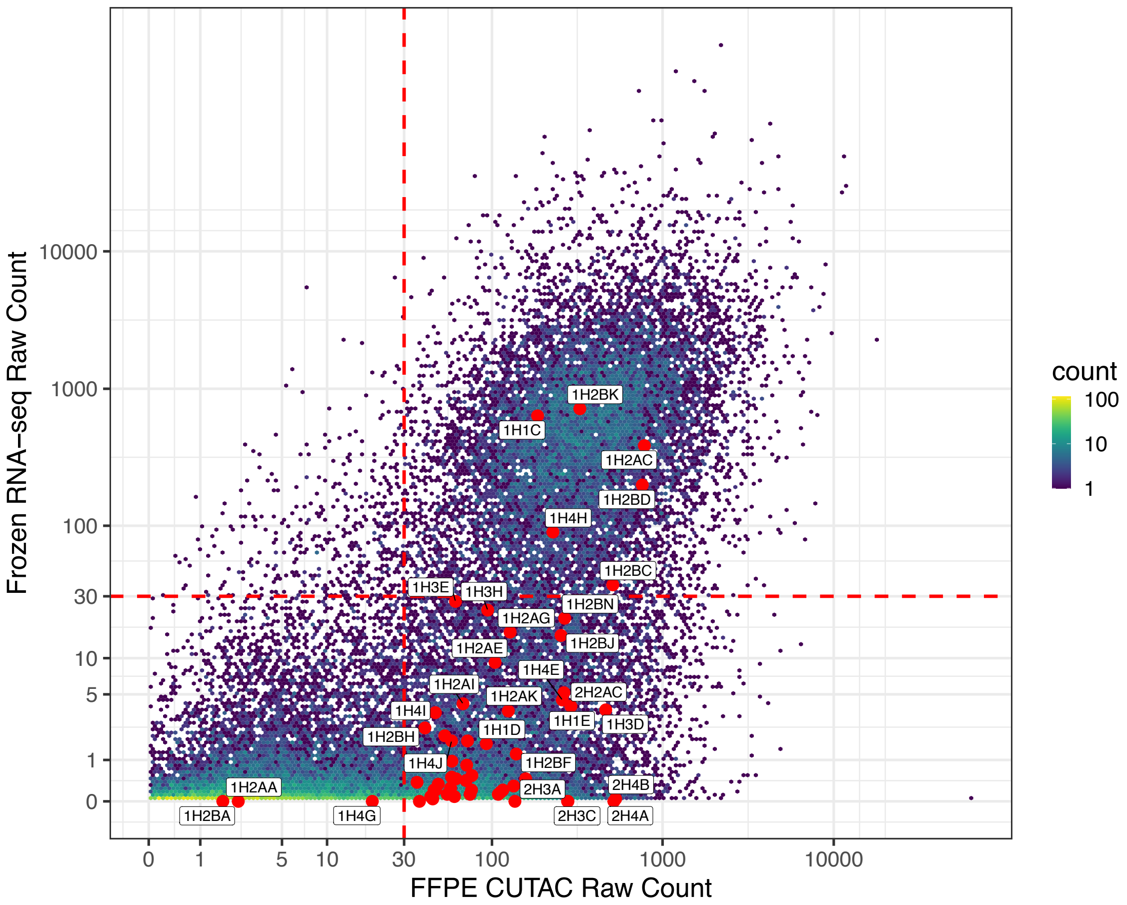


**Fig. S1. S-phase-dependent histone mRNAs are under-represented in meningioma patient RNA-seq data.** Log-scale hexbin plot compares the gene-by-gene distribution of average RNAPII fragment counts from FFPE-CUTAC to average RNA-seq transcript counts from frozen meningioma samples of the same patients. The individual histone gene signals are indicated as red circles, where the large majority are high in RNAPII but very low for RNA-seq. Exceptions in the upper right quadrant are likely to represent S-phase-independent replacement histones, including histone partners for H2A.X, H2A.Z, H3.3 and CENP-A variant histones, which are encoded by intron-containing genes outside of the S-phase-dependent histone clusters.

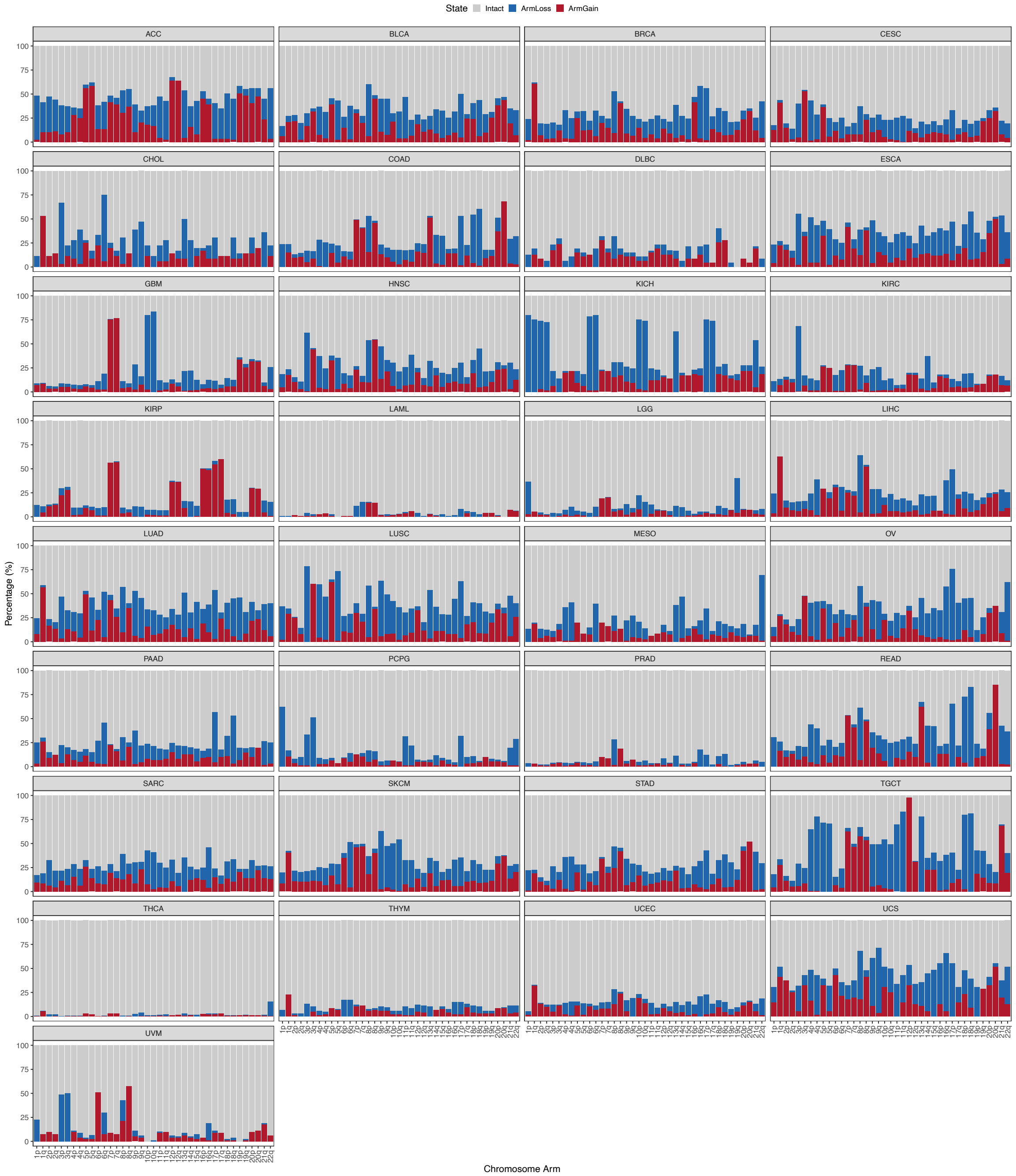


**Fig. S2. Percentage of whole-arm gains and losses in TCGA data.** See the legend in Figure 2.

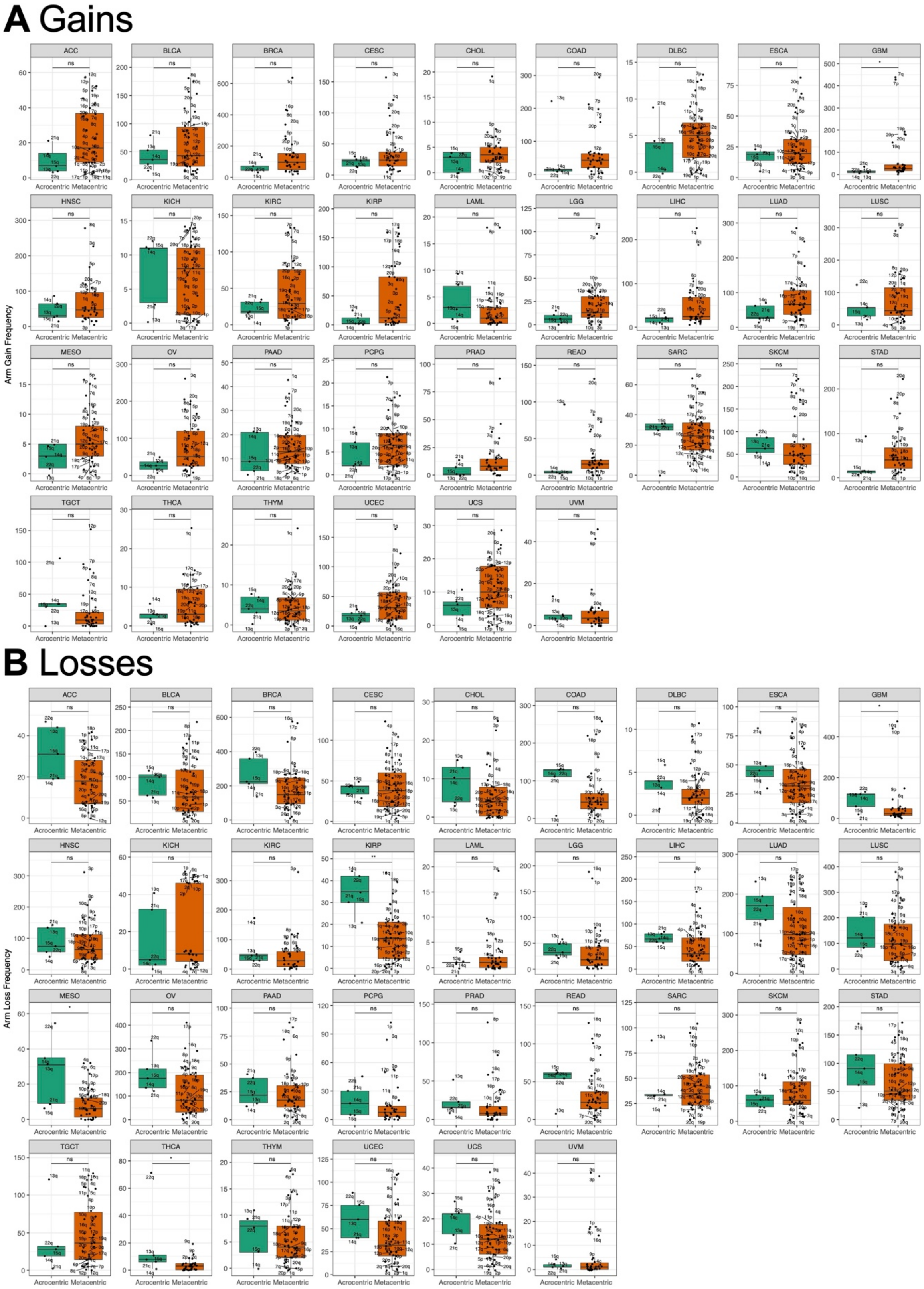
A Gains
Arm Gain Frequency
ACC
BLCA
BRCA
CESC
CHOL
COAD
DLBC
ESCA
GBM
HNSC
KICH
KIRC
KIRP
LAML
LGG
LIHC
LUAD
LUSC
MESO
OV
PAAD
PCPG
PRAD
READ
SARC
SKCM
STAD
TGCT
THCA
THYM
UCEC
UCS
UVM
Acrocentric Metacentric
B Losses
Arm Loss Frequency
Acrocentric Metacentric

# C Gains and losses

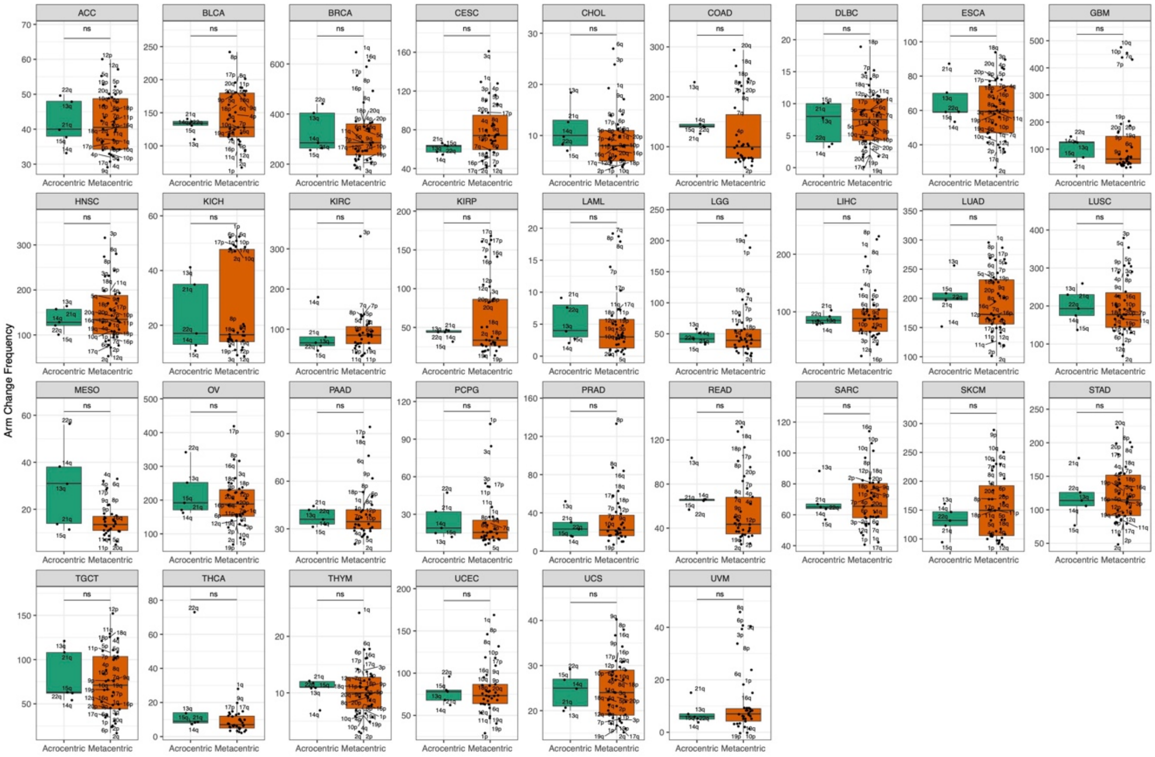


**Fig. S3: Acrocentric and metacentric whole-arm aneuploidies are recovered at similar frequencies in 33 cancer types.** We used whole-genome sequencing data from 10,674 cancer patients spanning 33 cancer types downloaded from The Cancer Genome Atlas (TCGA, https://portal.gdc.cancer.gov). Across all cancer types, we observed no significant differences in the frequencies of whole-arm gains and losses between acrocentric and metacentric chromosomes (Wilcoxon rank-sum test). For whole-arm gains, 32 of the 33 cancer types showed no significant difference, with the exception of glioblastoma multiforme (GBM), which exhibited a significant increase in gains driven by amplification of chromosome 7, a known glioblastoma cancer driver. In contrast, four cancer types, GBM, kidney renal papillary cell carcinoma (KIRP), mesothelioma (MESO), and thyroid carcinoma (THCA), demonstrated significant differences in whole-arm loss frequencies between acrocentric and metacentric chromosomes. Notably, the significant loss in GBM was driven by chromosome 10, another well-established cancer driver. Each dot represents a different autosomal chromosome arm (5 acrocentric long arms and 17 metacentrics). We inferred the chromosome arm gain or loss using ABSOLUTE [45] profiles for each patient and the aneuploidy results were directly retrieved from Table S2 of Taylor et al. [10]. (**A**) Gains; (**B**) Losses; (**C**) Gains or losses.

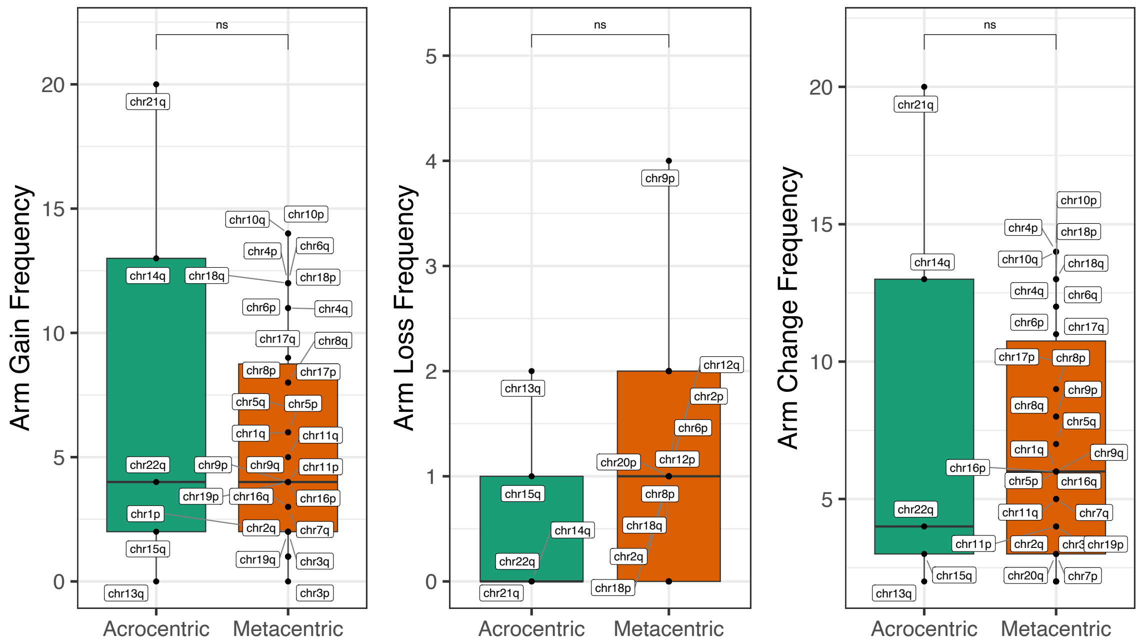


**Fig. S4: Acrocentric and metacentric whole-arm aneuploidies are recovered at similar frequencies in Nanopore long-read RNA-seq data.** See the legend in Figure 3. Data are from combined B-ALL and AML leukemia patient samples described in Ref (18).

Table S1. **Summary of whole-arm and whole-chromosome counts from TCGA data.** Source data is from Ref. [10] Table S2.

**Overall across 10,522 patient samples of 33 cancer types**

| | Totals | Mean | Median |
|---|---|---|---|
| Whole-arm | | | |
| Loss | 60833 | 1559.82 | 1705 |
| Gain | 44529 | 1141.77 | 859 |
| Per patient | | | |
| Loss | 5.78 | 0.148 | 0.162 |
| Gain | 4.23 | 0.108 | 0.082 |
| | Totals | Mean | Median |
| Whole-chromosome | | | |
| Loss | 15494 | 911 | 709 |
| Gain | 13526 | 795 | 529 |
| Per patient | | | |
| Loss | 1.47 | 0.087 | 0.067 |
| Gain | 1.29 | 0.076 | 0.05 |

**ACC**

| | Totals | Mean | Median |
|---|---|---|---|
| Whole-arm | | | |
| Loss | 798 | 20.46 | 19 |
| Gain | 830 | 21.28 | 15 |
| Per patient | | | |
| Loss | 8.97 | 0.23 | 0.21 |
| Gain | 9.33 | 0.24 | 0.17 |
| | Totals | Mean | Median |
| Whole-chromosome | | | |
| Loss | 273 | 16.06 | 16 |
| Gain | 363 | 21.35 | 13 |
| Per patient | | | |
| Loss | 3.07 | 0.18 | 0.18 |
| Gain | 4.08 | 0.24 | 0.15 |

**BLCA**

| | Totals | Mean | Median |
|---|---|---|---|
| Whole-arm | | | |
| Loss | 3034 | 77.79 | 65 |
| Gain | 2416 | 61.95 | 43 |
| Per patient | | | |
| Loss | 7.55 | 0.19 | 0.16 |

| | Totals | Mean | Median |
|---|---|---|---|
| Gain | 6.01 | 0.15 | 0.11 |
| | Totals | Mean | Median |
| Whole-chromosome | | | |
| Loss | 734 | 43.18 | 30 |
| Gain | 826 | 48.59 | 27 |
| Per patient | | | |
| Loss | 1.83 | 0.11 | 0.07 |
| Gain | 2.05 | 0.12 | 0.07 |

**BRCA**

| | Totals | Mean | Median |
|---|---|---|---|
| Whole-arm | | | |
| Loss | 7548 | 193.54 | 168 |
| Gain | 5147 | 131.97 | 94 |
| Per patient | | | |
| Loss | 7.2 | 0.18 | 0.16 |
| Gain | 4.91 | 0.13 | 0.09 |
| | Totals | Mean | Median |
| Whole-chromosome | | | |
| Loss | 1702 | 100.12 | 89 |
| Gain | 1312 | 77.18 | 62 |
| Per patient | | | |
| Loss | 1.62 | 0.1 | 0.08 |
| Gain | 1.25 | 0.07 | 0.06 |

**CESC**

| | Totals | Mean | Median |
|---|---|---|---|
| Whole-arm | | | |
| Loss | 1652 | 42.36 | 38 |
| Gain | 1348 | 34.56 | 24 |
| Per patient | | | |
| Loss | 5.58 | 0.14 | 0.13 |
| Gain | 4.55 | 0.12 | 0.08 |
| | Totals | Mean | Median |
| Whole-chromosome | | | |
| Loss | 419 | 24.65 | 18 |
| Gain | 326 | 19.18 | 15 |
| Per patient | | | |
| Loss | 1.42 | 0.08 | 0.06 |
| Gain | 1.1 | 0.06 | 0.05 |

**CHOL**

| | Totals | Mean | Median |
|---|---|---|---|

| | Totals | Mean | Median |
|---|---|---|---|
| Whole-arm | | | |
| Loss | 229 | 5.87 | 4 |
| Gain | 146 | 3.74 | 3 |
| Per patient | | | |
| Loss | 6.36 | 0.16 | 0.11 |
| Gain | 4.06 | 0.1 | 0.08 |
| | Totals | Mean | Median |
| Whole-chromosome | | | |
| Loss | 49 | 2.88 | 1 |
| Gain | 47 | 2.76 | 3 |
| Per patient | | | |
| Loss | 1.36 | 0.08 | 0.03 |
| Gain | 1.31 | 0.08 | 0.08 |

**COAD**

| | Totals | Mean | Median |
|---|---|---|---|
| Whole-arm | | | |
| Loss | 2669 | 68.44 | 45 |
| Gain | 2341 | 60.03 | 41 |
| Per patient | | | |
| Loss | 6.16 | 0.16 | 0.1 |
| Gain | 5.41 | 0.14 | 0.09 |
| | Totals | Mean | Median |
| Whole-chromosome | | | |
| Loss | 768 | 45.18 | 36 |
| Gain | 768 | 45.18 | 32 |
| Per patient | | | |
| Loss | 1.77 | 0.1 | 0.08 |
| Gain | 1.77 | 0.1 | 0.07 |

**DLBC**

| | Totals | Mean | Median |
|---|---|---|---|
| Whole-arm | | | |
| Loss | 115 | 2.95 | 2 |
| Gain | 183 | 4.69 | 4 |
| Per patient | | | |
| Loss | 2.45 | 0.06 | 0.04 |
| Gain | 3.89 | 0.1 | 0.09 |
| | Totals | Mean | Median |
| Whole-chromosome | | | |
| Loss | 23 | 1.35 | 2 |
| Gain | 78 | 4.59 | 4 |
| Per patient | | | |

| | | | |
|---|---|---|---|
| Loss | 0.49 | 0.03 | 0.04 |
| Gain | 1.66 | 0.1 | 0.09 |

**ESCA**

| | Totals | Mean | Median |
|---|---|---|---|
| Whole-arm | | | |
| Loss | 1447 | 37.1 | 34 |
| Gain | 930 | 23.85 | 19 |
| Per patient | | | |
| Loss | 8.88 | 0.23 | 0.21 |
| Gain | 5.71 | 0.15 | 0.12 |
| | Totals | Mean | Median |
| Whole-chromosome | | | |
| Loss | 330 | 19.41 | 18 |
| Gain | 226 | 13.29 | 10 |
| Per patient | | | |
| Loss | 2.02 | 0.12 | 0.11 |
| Gain | 1.39 | 0.08 | 0.06 |

**GBM**

| | Totals | Mean | Median |
|---|---|---|---|
| Whole-arm | | | |
| Loss | 2368 | 60.72 | 25 |
| Gain | 2305 | 59.1 | 23 |
| Per patient | | | |
| Loss | 4.16 | 0.11 | 0.04 |
| Gain | 4.05 | 0.1 | 0.04 |
| | Totals | Mean | Median |
| Whole-chromosome | | | |
| Loss | 786 | 46.24 | 17 |
| Gain | 1040 | 61.18 | 25 |
| Per patient | | | |
| Loss | 1.38 | 0.08 | 0.03 |
| Gain | 1.83 | 0.11 | 0.04 |

**HNSC**

| | Totals | Mean | Median |
|---|---|---|---|
| Whole-arm | | | |
| Loss | 3316 | 85.03 | 68 |
| Gain | 2486 | 63.74 | 44 |
| Per patient | | | |
| Loss | 6.48 | 0.17 | 0.13 |
| Gain | 4.86 | 0.12 | 0.09 |

| | Totals | Mean | Median |
|---|---|---|---|
| Whole-chromosome | | | |
| Loss | 671 | 39.47 | 34 |
| Gain | 532 | 31.29 | 24 |
| Per patient | | | |
| Loss | 1.31 | 0.08 | 0.07 |
| Gain | 1.04 | 0.06 | 0.05 |

**KICH**

| | Totals | Mean | Median |
|---|---|---|---|
| Whole-arm | | | |
| Loss | 692 | 17.74 | 8 |
| Gain | 274 | 7.03 | 8 |
| Per patient | | | |
| Loss | 10.65 | 0.27 | 0.12 |
| Gain | 4.22 | 0.11 | 0.12 |
| | Totals | Mean | Median |
| Whole-chromosome | | | |
| Loss | 294 | 17.29 | 8 |
| Gain | 116 | 6.82 | 8 |
| Per patient | | | |
| Loss | 4.52 | 0.27 | 0.12 |
| Gain | 1.78 | 0.1 | 0.12 |

**KIRC**

| | Totals | Mean | Median |
|---|---|---|---|
| Whole-arm | | | |
| Loss | 1913 | 49.05 | 30 |
| Gain | 1602 | 41.08 | 27 |
| Per patient | | | |
| Loss | 3.96 | 0.1 | 0.06 |
| Gain | 3.32 | 0.09 | 0.06 |
| | Totals | Mean | Median |
| Whole-chromosome | | | |
| Loss | 549 | 32.29 | 26 |
| Gain | 668 | 39.29 | 21 |
| Per patient | | | |
| Loss | 1.14 | 0.07 | 0.05 |
| Gain | 1.38 | 0.08 | 0.04 |

**KIRP**

| | Totals | Mean | Median |
|---|---|---|---|
| Whole-arm | | | |

| | | | |
|---|---|---|---|
| Loss | 657 | 16.85 | 16 |
| Gain | 1651 | 42.33 | 12 |
| Per patient | | | |
| Loss | 2.35 | 0.06 | 0.06 |
| Gain | 5.9 | 0.15 | 0.04 |
| | Totals | Mean | Median |
| Whole-chromosome | | | |
| Loss | 190 | 11.18 | 9 |
| Gain | 769 | 45.24 | 10 |
| Per patient | | | |
| Loss | 0.68 | 0.04 | 0.03 |
| Gain | 2.75 | 0.16 | 0.04 |

**LAML**

| | Totals | Mean | Median |
|---|---|---|---|
| Whole-arm | | | |
| Loss | 89 | 2.28 | 1 |
| Gain | 105 | 2.69 | 1 |
| Per patient | | | |
| Loss | 0.72 | 0.02 | 0.01 |
| Gain | 0.85 | 0.02 | 0.01 |
| | Totals | Mean | Median |
| Whole-chromosome | | | |
| Loss | 29 | 1.71 | 1 |
| Gain | 34 | 2 | 1 |
| Per patient | | | |
| Loss | 0.23 | 0.01 | 0.01 |
| Gain | 0.27 | 0.02 | 0.01 |

**LGG**

| | Totals | Mean | Median |
|---|---|---|---|
| Whole-arm | | | |
| Loss | 1270 | 32.56 | 19 |
| Gain | 751 | 19.26 | 11 |
| Per patient | | | |
| Loss | 2.46 | 0.06 | 0.04 |
| Gain | 1.45 | 0.04 | 0.02 |
| | Totals | Mean | Median |
| Whole-chromosome | | | |
| Loss | 247 | 14.53 | 8 |
| Gain | 264 | 15.53 | 9 |
| Per patient | | | |
| Loss | 0.48 | 0.03 | 0.02 |

| | | | |
|---|---|---|---|
| Gain | 0.51 | 0.03 | 0.02 |

**LIHC**

| | Totals | Mean | Median |
|---|---|---|---|
| Whole-arm | | | |
| Loss | 2097 | 53.77 | 42 |
| Gain | 1696 | 43.49 | 24 |
| Per patient | | | |
| Loss | 5.79 | 0.15 | 0.12 |
| Gain | 4.69 | 0.12 | 0.07 |
| | Totals | Mean | Median |
| Whole-chromosome | | | |
| Loss | 433 | 25.47 | 20 |
| Gain | 443 | 26.06 | 20 |
| Per patient | | | |
| Loss | 1.2 | 0.07 | 0.06 |
| Gain | 1.22 | 0.07 | 0.06 |

**LUAD**

| | Totals | Mean | Median |
|---|---|---|---|
| Whole-arm | | | |
| Loss | 4639 | 118.95 | 98 |
| Gain | 2934 | 75.23 | 62 |
| Per patient | | | |
| Loss | 9.22 | 0.24 | 0.19 |
| Gain | 5.83 | 0.15 | 0.12 |
| | Totals | Mean | Median |
| Whole-chromosome | | | |
| Loss | 1137 | 66.88 | 63 |
| Gain | 787 | 46.29 | 42 |
| Per patient | | | |
| Loss | 2.26 | 0.13 | 0.13 |
| Gain | 1.56 | 0.09 | 0.08 |

**LUSC**

| | Totals | Mean | Median |
|---|---|---|---|
| Whole-arm | | | |
| Loss | 4859 | 124.59 | 99 |
| Gain | 2911 | 74.64 | 46 |
| Per patient | | | |
| Loss | 10.08 | 0.26 | 0.21 |
| Gain | 6.04 | 0.15 | 0.1 |
| | Totals | Mean | Median |

| | Totals | Mean | Median |
|---|---|---|---|
| Whole-chromosome | | | |
| Loss | 1049 | 61.71 | 45 |
| Gain | 606 | 35.65 | 26 |
| Per patient | | | |
| Loss | 2.18 | 0.13 | 0.09 |
| Gain | 1.26 | 0.07 | 0.05 |

**MESO**

| | Totals | Mean | Median |
|---|---|---|---|
| Whole-arm | | | |
| Loss | 439 | 11.26 | 6 |
| Gain | 222 | 5.69 | 5 |
| Per patient | | | |
| Loss | 5.42 | 0.14 | 0.07 |
| Gain | 2.74 | 0.07 | 0.06 |
| | Totals | Mean | Median |
| Whole-chromosome | | | |
| Loss | 93 | 5.47 | 4 |
| Gain | 79 | 4.65 | 5 |
| Per patient | | | |
| Loss | 1.15 | 0.07 | 0.05 |
| Gain | 0.98 | 0.06 | 0.06 |

**OV**

| | Totals | Mean | Median |
|---|---|---|---|
| Whole-arm | | | |
| Loss | 4958 | 127.13 | 94 |
| Gain | 2758 | 70.72 | 40 |
| Per patient | | | |
| Loss | 8.98 | 0.23 | 0.17 |
| Gain | 5 | 0.13 | 0.07 |
| | Totals | Mean | Median |
| Whole-chromosome | | | |
| Loss | 1210 | 71.18 | 42 |
| Gain | 756 | 44.47 | 33 |
| Per patient | | | |
| Loss | 2.19 | 0.13 | 0.08 |
| Gain | 1.37 | 0.08 | 0.06 |

**PAAD**

| | Totals | Mean | Median |
|---|---|---|---|
| Whole-arm | | | |
| Loss | 960 | 24.62 | 18 |

| | | | |
|---|---|---|---|
| Gain | 588 | 15.08 | 13 |
| Per patient | | | |
| Loss | 5.78 | 0.15 | 0.11 |
| Gain | 3.54 | 0.09 | 0.08 |
| | Totals | Mean | Median |
| Whole-chromosome | | | |
| Totals | 254 | 14.94 | 11 |
| Loss | 172 | 10.12 | 10 |
| Gain | | | |
| Loss | 1.53 | 0.09 | 0.07 |
| Gain | 1.04 | 0.06 | 0.06 |

**PCPG**

| | Totals | Mean | Median |
|---|---|---|---|
| Whole-arm | | | |
| Loss | 661 | 16.95 | 8 |
| Gain | 262 | 6.72 | 6 |
| Per patient | | | |
| Loss | 4.03 | 0.1 | 0.05 |
| Gain | 1.6 | 0.04 | 0.04 |
| | Totals | Mean | Median |
| Whole-chromosome | | | |
| Loss | 135 | 7.94 | 3 |
| Gain | 87 | 5.12 | 5 |
| Per patient | | | |
| Loss | 0.82 | 0.05 | 0.02 |
| Gain | 0.53 | 0.03 | 0.03 |

**PRAD**

| | Totals | Mean | Median |
|---|---|---|---|
| Whole-arm | | | |
| Loss | 750 | 19.23 | 9 |
| Gain | 460 | 11.79 | 7 |
| Per patient | | | |
| Loss | 1.59 | 0.04 | 0.02 |
| Gain | 0.97 | 0.02 | 0.01 |
| | Totals | Mean | Median |
| Whole-chromosome | | | |
| Loss | 116 | 6.82 | 4 |
| Gain | 127 | 7.47 | 4 |
| Per patient | | | |
| Loss | 0.25 | 0.01 | 0.01 |
| Gain | 0.27 | 0.02 | 0.01 |

**READ**

| | Totals | Mean | Median |
|---|---|---|---|
| Whole-arm | | | |
| Loss | 1346 | 34.51 | 24 |
| Gain | 901 | 23.1 | 15 |
| Per patient | | | |
| Loss | 8.68 | 0.22 | 0.15 |
| Gain | 5.81 | 0.15 | 0.1 |
| | Totals | Mean | Median |
| Whole-chromosome | | | |
| Loss | 382 | 22.47 | 15 |
| Gain | 262 | 15.41 | 11 |
| Per patient | | | |
| Loss | 2.46 | 0.14 | 0.1 |
| Gain | 1.69 | 0.1 | 0.07 |

**SARC**

| | Totals | Mean | Median |
|---|---|---|---|
| Whole-arm | | | |
| Loss | 1595 | 40.9 | 34 |
| Gain | 1087 | 27.87 | 28 |
| Per patient | | | |
| Loss | 6.43 | 0.16 | 0.14 |
| Gain | 4.38 | 0.11 | 0.11 |
| | Totals | Mean | Median |
| Whole-chromosome | | | |
| Loss | 423 | 24.88 | 24 |
| Gain | 332 | 19.53 | 18 |
| Per patient | | | |
| Loss | 1.71 | 0.1 | 0.1 |
| Gain | 1.34 | 0.08 | 0.07 |

**SKCM**

| | Totals | Mean | Median |
|---|---|---|---|
| Whole-arm | | | |
| Loss | 3278 | 84.05 | 60 |
| Gain | 2646 | 67.85 | 49 |
| Per patient | | | |
| Loss | 7.11 | 0.18 | 0.13 |
| Gain | 5.74 | 0.15 | 0.11 |
| | Totals | Mean | Median |
| Whole-chromosome | | | |

| | Totals | Mean | Median |
|---|---|---|---|
| Loss | 1042 | 61.29 | 48 |
| Gain | 784 | 46.12 | 35 |
| Per patient | | | |
| Loss | 2.26 | 0.13 | 0.1 |
| Gain | 1.7 | 0.1 | 0.08 |

**STAD**

| | Totals | Mean | Median |
|---|---|---|---|
| Whole-arm | | | |
| Loss | 2734 | 70.1 | 61 |
| Gain | 2041 | 52.33 | 34 |
| Per patient | | | |
| Loss | 6.4 | 0.16 | 0.14 |
| Gain | 4.78 | 0.12 | 0.08 |
| | Totals | Mean | Median |
| Whole-chromosome | | | |
| Loss | 757 | 44.53 | 35 |
| Gain | 657 | 38.65 | 24 |
| Per patient | | | |
| Loss | 1.77 | 0.1 | 0.08 |
| Gain | 1.54 | 0.09 | 0.06 |

**TGCT**

| | Totals | Mean | Median |
|---|---|---|---|
| Whole-arm | | | |
| Loss | 1890 | 48.46 | 32 |
| Gain | 1010 | 25.9 | 10 |
| Per patient | | | |
| Loss | 12.19 | 0.31 | 0.21 |
| Gain | 6.52 | 0.17 | 0.06 |
| | Totals | Mean | Median |
| Whole-chromosome | | | |
| Loss | 718 | 42.24 | 25 |
| Gain | 270 | 15.88 | 5 |
| Per patient | | | |
| Loss | 4.63 | 0.27 | 0.16 |
| Gain | 1.74 | 0.1 | 0.03 |

**THCA**

| | Totals | Mean | Median |
|---|---|---|---|
| Whole-arm | | | |
| Loss | 215 | 5.51 | 3 |
| Gain | 194 | 4.97 | 3 |

| | Totals | Mean | Median |
|---|---|---|---|
| Per patient | | | |
| Loss | 0.46 | 0.01 | 0.01 |
| Gain | 0.41 | 0.01 | 0.01 |
| | Totals | Mean | Median |
| Whole-chromosome | | | |
| Loss | 47 | 2.76 | 1 |
| Gain | 74 | 4.35 | 2 |
| Per patient | | | |
| Loss | 0.1 | 0.01 | 0 |
| Gain | 0.16 | 0.01 | 0 |

**THYM**

| | Totals | Mean | Median |
|---|---|---|---|
| Whole-arm | | | |
| Loss | 224 | 5.74 | 4 |
| Gain | 183 | 4.69 | 4 |
| Per patient | | | |
| Loss | 2.11 | 0.05 | 0.04 |
| Gain | 1.73 | 0.04 | 0.04 |
| | Totals | Mean | Median |
| Whole-chromosome | | | |
| Loss | 76 | 4.47 | 3 |
| Gain | 65 | 3.82 | 3 |
| Per patient | | | |
| Loss | 0.72 | 0.04 | 0.03 |
| Gain | 0.61 | 0.04 | 0.03 |

**UCEC**

| | Totals | Mean | Median |
|---|---|---|---|
| Whole-arm | | | |
| Loss | 1652 | 42.36 | 36 |
| Gain | 1462 | 37.49 | 28 |
| Per patient | | | |
| Loss | 3.2 | 0.08 | 0.07 |
| Gain | 2.83 | 0.07 | 0.05 |
| | Totals | Mean | Median |
| Whole-chromosome | | | |
| Loss | 400 | 23.53 | 17 |
| Gain | 472 | 27.76 | 22 |
| Per patient | | | |
| Loss | 0.77 | 0.05 | 0.03 |
| Gain | 0.91 | 0.05 | 0.04 |

**UCS**

| | Totals | Mean | Median |
|---|---|---|---|
| Whole-arm | | | |
| Loss | 558 | 14.31 | 14 |
| Gain | 406 | 10.41 | 9 |
| Per patient | | | |
| Loss | 9.96 | 0.26 | 0.25 |
| Gain | 7.25 | 0.19 | 0.16 |
| | Totals | Mean | Median |
| Whole-chromosome | | | |
| Loss | 111 | 6.53 | 5 |
| Gain | 113 | 6.65 | 7 |
| Per patient | | | |
| Loss | 1.98 | 0.12 | 0.09 |
| Gain | 2.02 | 0.12 | 0.12 |

**UVM**

| | Totals | Mean | Median |
|---|---|---|---|
| Whole-arm | | | |
| Loss | 181 | 4.64 | 1 |
| Gain | 253 | 6.49 | 4 |
| Per patient | | | |
| Loss | 2.26 | 0.06 | 0.01 |
| Gain | 3.16 | 0.08 | 0.05 |
| | Totals | Mean | Median |
| Whole-chromosome | | | |
| Loss | 47 | 2.76 | 0 |
| Gain | 71 | 4.18 | 3 |
| Per patient | | | |
| Loss | 0.59 | 0.03 | 0 |
| Gain | 0.89 | 0.05 | 0.04 |
